# A β-Arrestin 2-Biased Dopamine Receptor Type 2 (DRD2) Agonist Is More Efficacious Than Cabergoline in Reducing Cell Proliferation in PRL-Secreting but Not in Non-Functioning Pituitary Tumor Cells

**DOI:** 10.3390/cancers15123218

**Published:** 2023-06-16

**Authors:** Genesio Di Muro, Federica Mangili, Emanuela Esposito, Anna Maria Barbieri, Rosa Catalano, Donatella Treppiedi, Giusy Marra, Emma Nozza, Andrea G. A. Lania, Emanuele Ferrante, Marco Locatelli, Maura Arosio, Erika Peverelli, Giovanna Mantovani

**Affiliations:** 1 Department of Clinical Sciences and Community Health, University of Milan, 20122 Milan, Italy; genesio.dimuro@uniroma1.it (G.D.M.); giovanna.mantovani@unimi.it (G.M.); 2 Department of Experimental Medicine, University Sapienza of Rome, 00100 Rome, Italy; 3 Endocrinology Unit, Fondazione Istituto di Ricovero e Cura a Carattere Scientifico (IRCCS) Ca’ Granda Ospedale Maggiore Policlinico, 20122 Milan, Italy; federica.mangili@policlinico.mi.it (F.M.);; 4 PhD Program in Experimental Medicine, University of Milan, 20100 Milan, Italy; 5 Department of Biomedical Sciences, Humanitas University, 20090 Pieve Emanuele, Italy; 6 Endocrinology and Diabetology Unit, Istituto di Ricovero e Cura a Carattere Scientifico (IRCCS) Humanitas Research Hospital, 20089 Rozzano, Italy; 7 Neurosurgery Unit, Fondazione Istituto di Ricovero e Cura a Carattere Scientifico (IRCCS) Ca’ Granda Ospedale Maggiore Policlinico, 20122 Milan, Italy; 8 Department of Pathophysiology and Transplantation, University of Milan, 20122 Milan, Italy

**Keywords:** dopamine receptor type 2, pituitary tumors, beta arrestin 2

## Abstract

**Simple Summary:**

Dopamine receptor type 2 (DRD2) mediates antitumoral effects in different types of pituitary tumors with variable efficacy. DRD2′s functionally selective agonists are able to preferentially activate the canonical G-protein-mediated signaling arm or the β-arrestin 2 pathway, which have recently been implicated in antimitotic signaling of DRD2. In a PRL-secreting pituitary tumor cell line, the β-arrestin 2-biased DRD2 ligand UNC9994 induced a stronger reduction in cell proliferation compared to the unselective agonist cabergoline, the currently used drug for this type of tumor. Primary PRL-secreting human tumoral cells that were resistant to cabergoline showed good reduction in cell proliferation and hormone secretion after UNC9994 incubation. Overall, these data suggest that UNC9994 may represent an alternative pharmacological strategy for cabergoline-resistant or poorly responsive tumors. In contrast, this strategy is not beneficial for non-functioning pituitary tumors.

**Abstract:**

The molecular events underlying the variable effectiveness of dopamine receptor type 2 (DRD2) agonists in pituitary neuroendocrine tumors (PitNETs) are not known. Besides the canonical pathway induced by DRD2 coupling with Gi proteins, the β-arrestin 2 pathway contributes to DRD2′s antimitotic effects in PRL- and NF-PitNETs. A promising pharmacological strategy is the use of DRD2-biased agonists that selectively activate only one of these two pathways. The aim of the present study was to compare the effects of two biased DRD2 ligands, selectively activating the G protein (MLS1547) or β-arrestin 2 (UNC9994) pathway, with unbiased DRD2 agonist cabergoline in PRL- and NF-PitNET cells. In rat tumoral pituitary PRL-secreting MMQ cells, UNC9994 reduced cell proliferation with a greater efficacy compared to cabergoline (−40.2 ± 20.4% vs. −21 ± 10.9%, *p* < 0.05), whereas the G-protein-biased agonist induced only a slight reduction. β-arrestin 2 silencing, but not pertussis toxin treatment, reverted UNC9994 and cabergoline’s antiproliferative effects. In a cabergoline-resistant PRL-PitNET primary culture, UNC9994 inhibited cell proliferation and PRL release. In contrast, in NF-PitNET primary cultures (*n* = 23), biased agonists did not show better antiproliferative effects than cabergoline. In conclusion, the preferential activation of the β-arrestin 2 pathway by UNC9994 improves DRD2-mediated antiproliferative effects in PRL-PitNETs, suggesting a new pharmacological approach for resistant or poorly responsive tumors.

## 1. Introduction

Dopamine receptor type 2 (DRD2) agonists (DAs) exert antitumoral effects in different types of pituitary neuroendocrine tumors (PitNETs), with highly variable therapeutic responses. DAs, especially cabergoline, are the first-choice treatment for patients with prolactin (PRL)-secreting PitNETs, since they are effective in inducing prolactin normalization and in reducing tumor size, but about 10% of patients are resistant to the treatment [1]. In non-functioning (NF)-PitNETs, DAs can reduce tumor mass in a subset of patients, but their use is not recommended, since published data are insufficient and controversial in demonstrating their efficacy [2,3,4,5,6].

The molecular mechanisms underlying DA resistance in PitNET have been only partially identified [7]. The reasons for the different responsiveness levels of PitNETs to DAs are not limited to the expression levels of DRD2 in tumoral cells but also involve the intracellular events that specifically occur downstream of DRD2 activation in the different types of pituitary cells.

DRD2 is the predominantly expressed dopamine receptor isoform in normal and tumoral pituitary cells. It belongs to the D2-like dopamine receptor subfamily, which includes inhibitory heterotrimeric G-protein-coupled receptors DRD2, DRD3 and DRD4 [8]. The G-protein-mediated pathway, which leads to adenylyl cyclase inhibition and consequent intracellular cyclic adenosine monophosphate (cAMP) reduction, is the best-studied DRD2 signal transduction pathway, but the existence of G-protein-independent DRD2 signaling is now well-documented. This pathway is initiated by GPCR-kinase (GRK)-dependent phosphorylation of DRD2 and the subsequent recruitment of the multifunctional adaptor protein β-arrestin 2 [9,10,11].

We recently found that DRD2 requires β-arrestin 2 pathway activation for transduction of the antiproliferative signals in PRL- and NF-PitNETs [12]. β-arrestin 2 recruitment to DRD2 leads to the formation of a β-arrestin-dependent complex, including AKT and protein phosphatase 2a, which results in the dephosphorylation of AKT [11,13], leading to the inhibition of cell proliferation. In tumoral lactotrophs and NF-PitNETs, the lack of β-arrestin 2 prevents the inhibitory effect of DRD2 on AKT, with a consequent resistance to the antimitotic action of DAs [12].

It is known that G protein pathway-mediated cAMP reduction, together with decreased intracellular calcium levels due to inhibition of phosphatidylinositol metabolism and inward calcium currents, results in decreased PRL secretion. However, the activation of the cAMP pathway exerts an antiproliferative effect in both NF-PitNETs and PRL-secreting tumor cells [14,15]. cAMP suppression induced by G protein signaling might thus weaken the antiproliferative effects of DAs in NF-and PRL-PitNETs. However, the differential contribution of the two signaling pathways in the therapeutic effects of cabergoline in the different types of PitNET remains unclear.

Like many other GPCRs, DRD2 exhibits functional selectivity due to the ability to adopt multiple conformations, leading to specific signaling through a subset of its normal multiple pathways [16]. Selective targeting of one specific signaling arm may improve the therapeutic efficacies and side-effect profiles of DRD2-related drugs.

Different DRD2 ligands that exhibit functional selectivity (biased agonists) have been characterized so far. In particular, UNC9994 is a functionally selective β-arrestin-biased DRD2 ligand based on the chemical scaffold of aripiprazole. It has essentially no activity in the G protein pathway but retains partial agonism in the β-arrestin 2 pathway [17]. On the other hand, MLS1547 is a highly efficacious agonist at DRD2-mediated, G-protein-linked signaling and antagonizes dopamine-stimulated β-arrestin 2 recruitment to DRD2 [18].

In the present study, we compared the functional effects of cabergoline, UNC9994 and MLS1547 in PRL- and NF-PitNET cells to evaluate the contributions of the G protein and β-arrestin signaling pathways to the antitumoral effects of DRD2, with the aim to improve the efficacy of DRD2-targeting drugs in PitNETs.

## 2. Materials and Methods

### 2.1. Cell Cultures

PRL-secreting rat tumoral pituitary MMQ cells (ATCC CRL-10609) were cultured in an RPMI medium (Life Technologies, ThermoFisher, Carlsbad, CA, USA) with 15% horse serum, 2.5% fetal bovine serum (FBS) (both from Gibco, Invitrogen, Life Technologies Inc., Carlsbad, CA, USA) and 2 mM of glutamine and antibiotics. Primary cultured PitNET cells were obtained from surgically removed human tumors (23 patients with NF-PitNETs and 2 with PRL-PitNETs), as has been previously described [12]. Cells were maintained in a DMEM medium (Gibco, Invitrogen, Life Technologies Inc., Carlsbad, CA, USA) supplemented with 10% FBS and 2 mM of glutamine and antibiotics. The NF-PitNET primary cultures were checked daily with visual inspection with an optical microscope to exclude fibroblast contamination.

This study (#1167_2022) was approved by the local Ethics Committee (Comitato Etico Milano Area 2), and each patient gave informed consent.

### 2.2. Proliferation Assay

Colorimetric measurement of 5-bromo-2-deoxyuridine (BrdU) incorporated during DNA synthesis was performed [19]. In particular, primary cultured (5 × 10^4^ cells/well) or MMQ (2 × 10^4^ cells/well) cells were seeded in polylysine-coated 96-well plates in a starved medium. The cells were incubated for 72 h at 37 °C with cabergoline (Sigma-Aldrich, St. Louis, MO, USA), UNC9994 (Axon Medchem, GZ Groningen, The Netherlands) or MLS1547 (Sigma-Aldrich, St. Louis, MO, USA), in the indicated doses, in the complete medium. BrdU incorporation was allowed in the newly synthesized DNA for 24 h (primary cultures) or 2 h (MMQ).

### 2.3. Western Blot Analysis

In total, 60 μg of proteins extracted from treated cells were analyzed. The primary antibodies used were p-AKT (Ser473), total AKT and cyclin D3 (1:1000), as well as β-arrestin 2 (1:2000). The secondary antibodies used were anti-rabbit and anti-mouse (1:2000) (All Cell Signaling, Denvers, MA, USA). Anti-GAPDH was used as housekeeping (1:4000) (Ambion, Life Technologies, Thermo-Fisher, Carlsbad, CA, USA). Chemiluminescence was detected with a ChemiDOC-IT Imaging System (UVP, Upland, CA, USA) and analyzed with NIH ImageJ 1.52t software.

### 2.4. β-Arrestin 2 Silencing

MMQ cells were transfected with β-arrestin 2 SMARTpool siRNAs for 72 h at 37 °C (Dharmacon, GE Healthcare Life Sciences, Chicago, IL, USA) [12]. Silencing efficiency was checked with western blot analysis.

### 2.5. Pertussis Toxin Pretreatment

To perform western blot experiments and proliferation assays, we incubated the cells overnight at 37 °C with 100 ng/μL of pertussis toxin (PTX) (Invitrogen, Carlsbad, CA, USA). After PTX pretreatment, DRD2 agonists (cabergoline, UNC9994 or MLS1547) were added into the cell medium and left for 10 min (western blot) or 72 h (proliferation assay).

### 2.6. PRL Secretion Assay

Specific Elisa immunoassay kits were used to detect rat PRL levels in MMQ cell culture media, following the manufacturer’s instructions (Fine Test, Wuhan Fine Biotech Co., Ltd., Wuhan, China). Briefly, after 24 h of incubation with 100 nM treatments (Cabergoline, UNC9994, MLS1547), culture media were collected and the assay performed (three times, in triplicate). Normalization was performed, as has been described before [20]. PRL hormone levels of human derived PRL-PitNET cultured cells were measured in a culture medium, collected after 24 h treatment incubation, with a specific chemiluminescent immunometric assay (Elecsys Prolactin II, Roche Diagnostics GmbH, Mannheim, Germany).

### 2.7. RT-PCR and Real-Time RT-PCR Analysis

The total RNA extracted from the frozen tissue was reverse-transcribed (RevertAid H Minus First Strand cDNA Synthesis Kit, Thermo Fisher Scientific, Waltham, MA, USA), and the cDNA was amplified with specific primers for *SF1* and *PIT1* (Supplementary Tables S1 and S2).

Real time RT-PCR was carried out with SsoFast^TM^ EvaGreen^®^ Supermix (Bio-Rad Laboratories, Hercules, CA, USA) in a QuantStudio™ 3 Real-Time PCR System (Applied Biosystems, Thermo Fisher Scientific, Waltham, MA, USA). The primers used for the *DRD2* isoforms and *β-arrestin 2* are listed in Supplementary Table S1. Data were analyzed with QuantStudio^TM^ Design & Analysis Software v1.5.1, using the ΔCt method.

### 2.8. Statistical Analysis

The results are expressed as means ± SD. The significance between any 2 series of data was determined using a two-tailed Student *t*-test. To evaluate the gene expression differences in the NF-PitNET tissues, Kruskal–Wallis’ and Dunn’s post hoc tests were applied. *p* < 0.05 was considered statistically significant.

## 3. Results

### 3.1. DRD2-Biased Agonists’ Effects on Cell Proliferation and PRL Secretion in MMQ Cells

**Figure 1:**
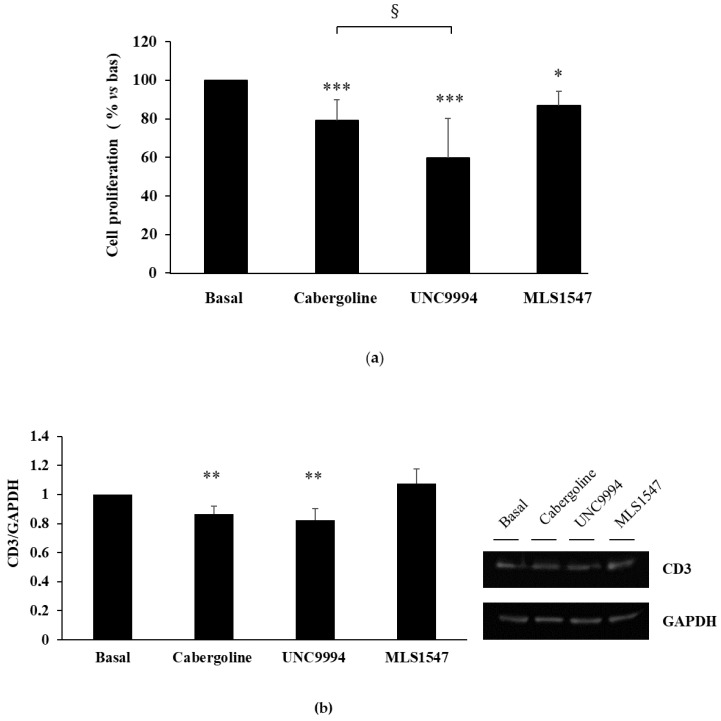

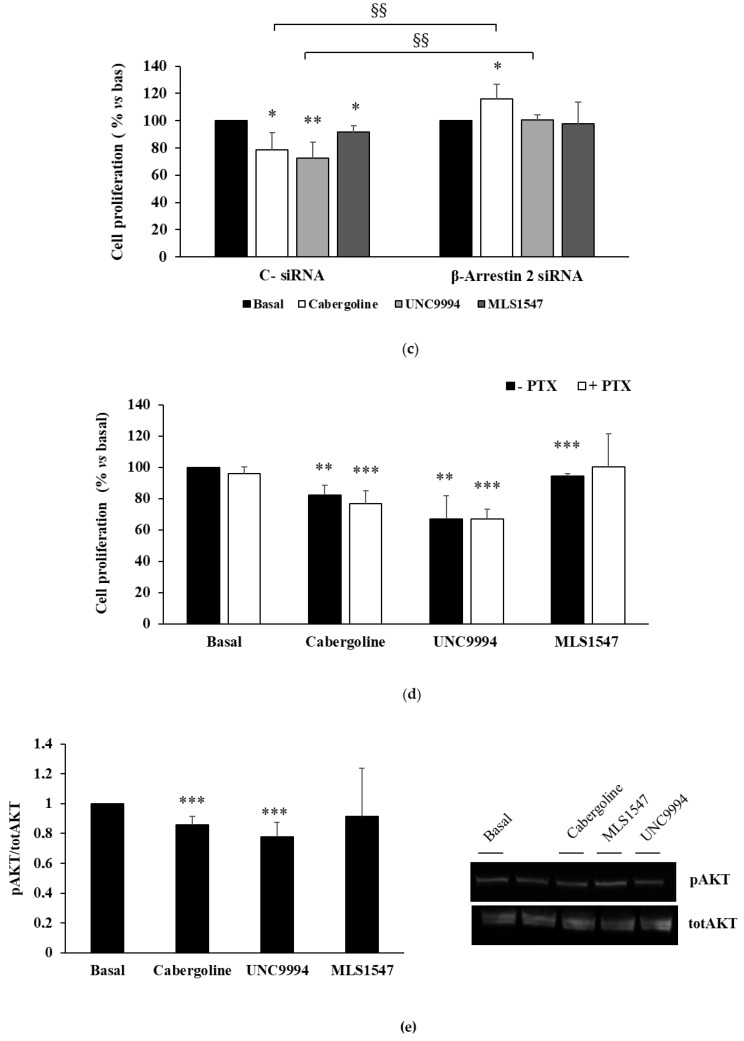

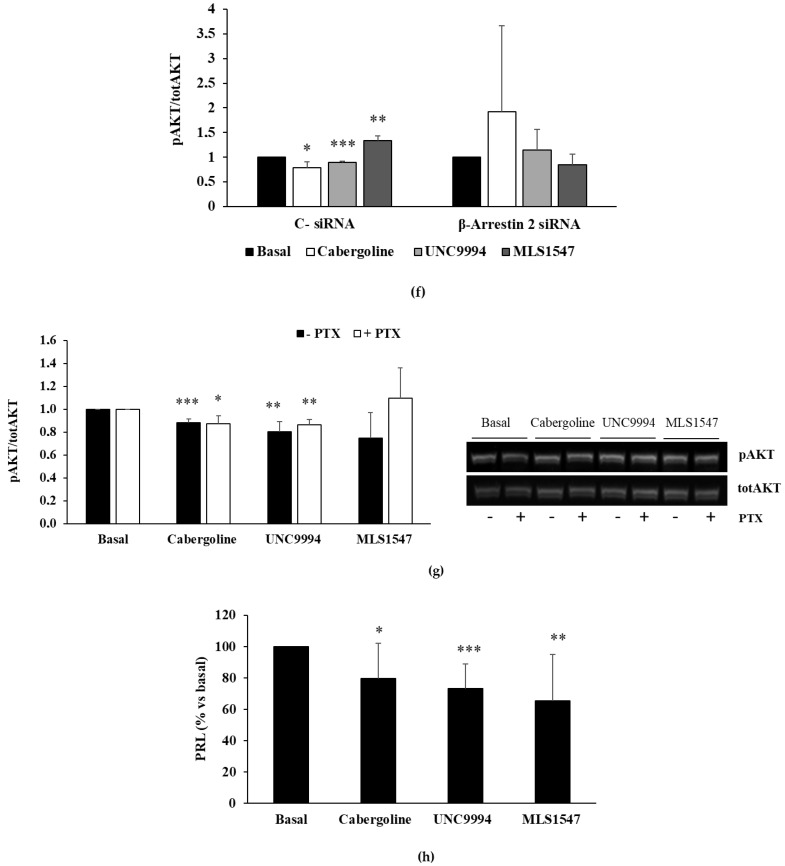
DRD2 biased agonists’ effects on cell proliferation and PRL secretion in MMQ cells. (**a**) MMQ cells were cultured with 100 nM cabergoline, 100 nM UNC9994, 100 nM MLS1547, and each determination was carried out in triplicate. (**b**) This graph shows the quantification of cyclin D3 levels normalized to GAPDH after a 3 h treatment incubation. A representative immunoblot is shown, original blot see Supplementary File S1. (**c**) MMQ cells were transiently transfected with β-arrestin 2 siRNA or negative control siRNA (C−), then incubated with treatments, and BrdU incorporation in newly synthesized DNA was measured. (**d**) MMQ cells were pretreated overnight with PTX, then incubated with treatments, and BrdU incorporation was measured. (**e**) MMQ cells were incubated for 10 min with treatments. This graph shows the quantification of p-AKT on Ser473, normalized to total AKT. A representative immunoblot is shown. (**f**) MMQ cells were transfected with β-arrestin 2 or negative control siRNAs (C−) and then incubated for 10 min with treatments. This graph shows the quantification of p-AKT normalized to total AKT. (**g**) MMQ cells were pretreated overnight with PTX, then incubated for 10 min with treatments. This graph shows the quantification of p-AKT on Ser473, normalized to total AKT. A representative immunoblot is shown. (**h**) MMQ cells were incubated with or without treatments, and PRL levels in cell culture media were measured. This graph shows the percentages of PRL levels compared to basal. Values represent means (±S.D.), normalized vs. respective basal. All the experiments were replicated at least three times. * = *p* < 0.05; ** = *p* < 0.01; *** = *p* < 0.001 vs. corresponding basal; § = *p* < 0.05; §§ = *p* < 0.01 vs. corresponding C− treated cells.

To investigate the contributions of the G protein and β-arrestin 2 pathways in mediating DRD2 antiproliferative effects in tumoral lactotrophs, we incubated PRL-secreting rat MMQ cells with cabergoline at 100 nM [21] or with one of the two biased DRD2 ligands, UNC9994 and MLS1547, at the same dose. We found that the UNC9994 reduced cell proliferation with a greater efficacy compared to the cabergoline (−40.2 ± 20.4% vs. −21 ± 10.9%, *p* < 0.05 vs. cabergoline), whereas the MLS1547 treatment resulted in a negligible effect (−13.2 ± 7.4%, *p* < 0.05 vs. bas) (Figure 1a).

Consistently with the results for cell proliferation, only the cabergoline and UNC9994 treatments resulted in reduction in cyclin D3 expression (−13.4 ± 5.4% and −17.6 ± 7.8%, respectively; both *p* < 0.01 vs. bas) (Figure 1b).

To test the engagement of β-arrestin 2 in the observed effects, we performed genetic silencing experiments in the MMQ cells. We found that the antiproliferative effects of both cabergoline and UNC9994 depend on the presence of β-arrestin 2, since they were completely reverted in β-arrestin 2-silenced MMQ cells (Figure 1c). In contrast, pertussis toxin (PTX) pretreatment did not affect the efficacy of the cabergoline or the UNC9994 in reducing cell proliferation, suggesting that PTX-sensitive inhibitory G proteins are not involved in the antimitotic action of these compounds (Figure 1d). On the contrary, the slight effect of the MLS1547 on cell proliferation was completely abolished by the PTX pretreatment, suggesting involvement of the Gi protein pathway. To assess the ability of DRD2-biased agonists to reduce PRL secretion levels, MMQ cells were treated with cabergoline, UNC9994 and MLS1547 for 24 h, and the PRL was measured in the cell culture media. Our results show that the cabergoline and both biased ligands reduced PRL secretion with comparable efficacy (cabergoline: −20.3 ± 22.3%, *p* < 0.05 vs. bas; UNC9994: −26.7 ± 15.5%, *p* < 0.001 vs. bas; MLS1547: −34.7 ± 29.5%, *p* < 0.01 vs. bas) (Figure 1h). Since the β-arrestin 2-mediated pathway activation induced a dephosphorylation of AKT on the residue Ser473 in the MMQ cells [12], we tested the effects of biased agonists on the AKT Ser473 phosphorylation levels (pAKT). As expected, both the cabergoline and the UNC9994 reduced AKT phosphorylation (−14.1 ± 5.4%; −22.2 ± 9.7%, respectively; both *p* < 0.001 vs. bas), whereas the MLS1547 did not change the AKT phosphorylation levels (Figure 1e). As expected, β-arrestin 2 silencing abolished the cabergoline’s and the UNC9994′s effects on the AKT phosphorylation (Figure 1f). On the contrary, PTX pretreatment did not affect the ability of the cabergoline or the UNC9994 to dephosphorylate the AKT (Figure 1g).

### 3.2. DRD2-Biased Agonists’ Effects on Cell Proliferation and PRL Secretion in Primary Cultured PRL-PitNET Cells

We then compared cabergoline’s and UNC9994′s effects in primary cell cultures derived from two surgically removed human PRL-PitNETs. Due to the low number of viable cells obtained from the tumors’ dispersion, we chose to test only the β-arrestin 2-biased agonist, based on the promising results obtained with the MMQ cells. We considered a tumor responsive to treatment when the drug reduced cell proliferation of at least 15%.

**Figure 2:**
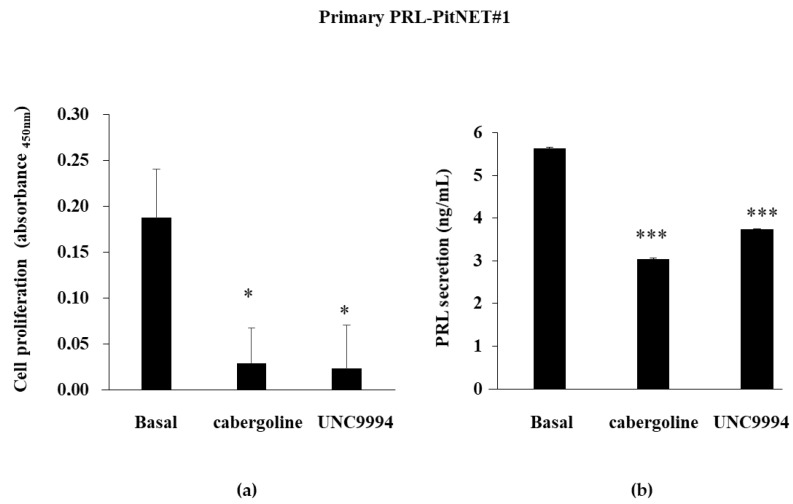

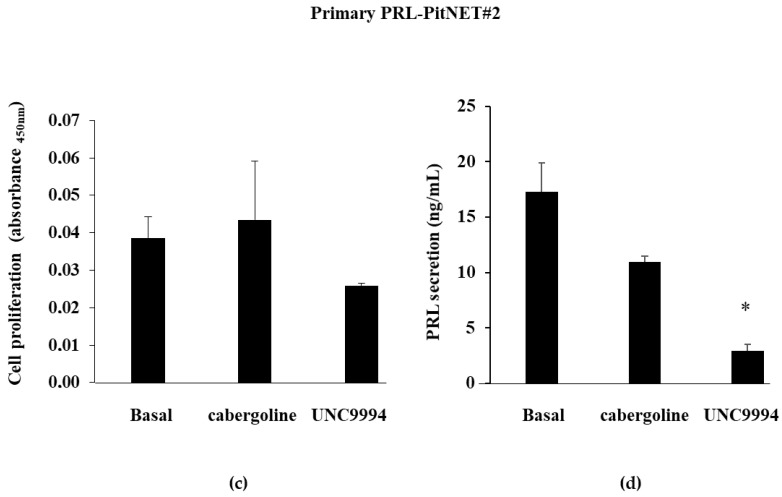
Cabergoline’s and UNC9994′s effects on cell proliferation and PRL secretion in primary cultured PRL-PitNET cells. Cells cultured from 2 PRL-PitNETs were incubated for 72 h at 37 °C with 100 nM cabergoline and 100 nM UNC9994. (**a**,**c**) BrdU incorporation in newly synthesized DNA or (**b**,**d**) PRL levels were measured. Each determination was carried out in triplicate. The graphs show means ± SD. * *p* < 0.05, *** *p* < 0.001 vs. corresponding basal.

PRL-PitNET #1 was responsive in vitro to the cabergoline’s antiproliferative action (−84.4 ± 21.3%), and the UNC9994 treatment induced a comparable reduction in cell proliferation (−87.6 ± 27.4%) (Figure 2a). In this tumor, both DRD2 agonists were effective in reducing PRL secretion in the cell culture media (cabergoline: −46.14 ± 1.2%; UNC9994: −33.7 ± 0.57%; both *p* < 0.001 vs. bas) (Figure 2b).

On the contrary, PRL-PitNET #2 was resistant in vitro to the cabergoline’s inhibitory effects on both cell proliferation and PRL secretion but responsive to the UNC9994. Indeed, the UNC9994 incubation reduced both cell proliferation (−33 ± 0.5%) and PRL secretion (−83 ± 19.8%, *p* < 0.05) (Figure 2c,d).

### 3.3. DRD2-Biased Agonists’ Effects on Cell Proliferation in Primary Cultured NF-PitNET Cells

**Figure 3:**
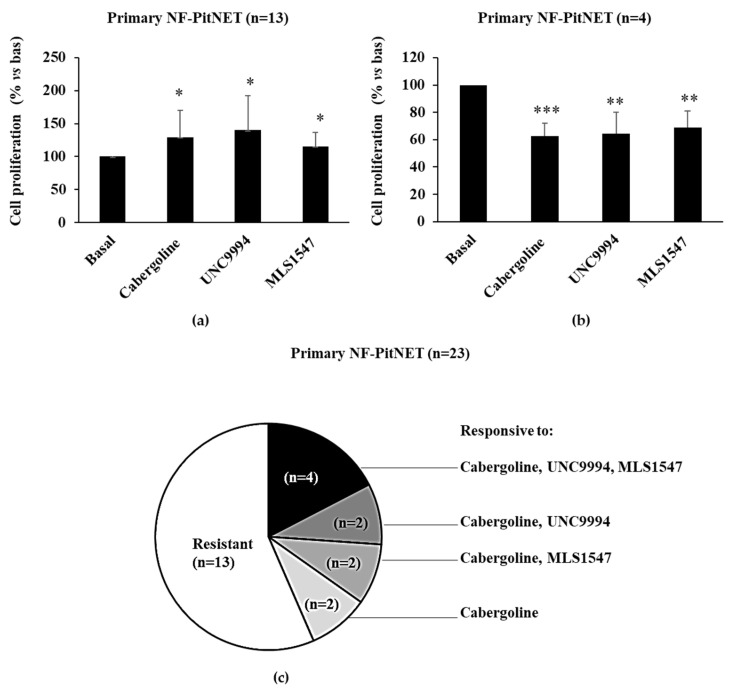
DRD2 ligands’ antimitotic effects in primary cultured NF-PitNET cells. Cells cultured from NF-PitNETs were incubated for 72 h at 37 °C with 100 nM cabergoline, 100 nM UNC99 94 and 100 nM MLS1547. BrdU incorporation in newly synthesized DNA was measured, and each determination was carried out in triplicate. * *p* < 0.05, ** *p* < 0.01, *** *p* < 0.001 vs. corresponding basal. The graphs represent the means of treatment-resistant (**a**) and sensitive (**b**) primary cultured NF-PitNET cells, respectively. The pie chart (**c**) summarizes the number of tumors responsive to different treatments.

The antiproliferative effects of selective DRD2 agonists were also tested on primary cultures derived from surgically removed NF-PitNETs. As for PRL-PitNETs, we considered a tumor responsive when the treatment reduced cell proliferation by at least 15%. We found that thirteen of the twenty-three primary cultures were resistant to the antimitotic action of all DRD2 ligands tested (Figure 3a,c) and four were responsive to all the agonists to a similar extent (Figure 3b,c). In the remaining tumors, we found great heterogeneity: two NF-PitNETs were responsive only to cabergoline and UNC9994, two only to cabergoline and MLS1547 and two only to cabergoline (Figure 3c). Overall, 10 NF-PitNETs were responsive to at least the cabergoline. These data suggest that in NF-PitNETs, DRD2 can reduce cell proliferation by both the β-arrestin 2 and the G-protein-mediated pathways, depending on the tumor, and that in tumors resistant to all DRD2 ligands, alterations common to the two pathways may be present. In resistant PitNETs, DRD2 agonists even increased cell proliferation, as has been previously observed [12].

The qRT-PCR experiment showed no differences in DRD2 long-and short-isoform or β-arrestin 2 expression in NF-PitNETs belonging to different groups (Supplementary Figure S1). In addition, we ruled out a role of different lineage derivation in determining responsiveness to DRD2 agonists in terms of cell proliferation. The expression of *SF1*, a marker of gonadotroph cell lineage [22], was detected with RT-PCR in all NF-PitNETs tested, regardless of DRD2 ligand responsiveness, except for in three tumors that were resistant to all DRD2 ligands; one out of these was positive for *PIT1* (Supplementary Figure S2a,b).

## 4. Discussion

In the present paper, we have explored the possibility of selectively activating a specific DRD2-mediated signal transduction pathway in PitNET cells, with the aim to improve desired biological effects, e.g., reduction in cell proliferation and, for PRL-PitNETs, hormone secretion.

Two DRD2-biased agonists that selectively activate the canonical G-protein-mediated pathway (MLS1547) or the non-canonical β-arrestin 2-mediated pathway (UNC9994) have been well-characterized [17,18].

Although the inhibitory G protein pathway has been the most studied and linked to hormone secretion regulation, we recently showed that DRD2 activation of the β-arrestin 2 pathway leads to cell proliferation inhibition by AKT dephosphorylation in PRL- and NF-PitNETs [12]. In particular, in MMQ cells, the inhibitory effects of DRD2 agonist BIM53097 on cell proliferation and AKT dephosphorylation were abolished in cells silenced for β-arrestin 2, but not in cells pretreated with PTX [12], which inactivates inhibitory G proteins by catalyzing the ADP-ribosylation of a cysteine residue on the carboxy terminal of the α subunits of Gi and Go.

In the present work, β-arrestin 2 silencing and PTX pretreatment experiments also demonstrated that cabergoline’s inhibitory effects on cell proliferation and AKT phosphorylation in MMQ cells are mediated by β-arrestin 2, with no role played by Gi/o proteins. Interestingly, the DRD2-biased agonist UNC9994 was more effective than cabergoline in reducing cell proliferation. We can hypothesize that its effectiveness is due to the activation of the β-arrestin pathway, with consequent reduction in AKT phosphorylation, associated with a failure to reduce intracellular cAMP levels. Indeed, in PRL-PitNETs, cAMP plays an inhibitory role in cell proliferation [14], and the inhibitory G protein pathway activated by cabergoline, leading to cAMP reduction, might counteract the antiproliferative effect induced by β-arrestin 2.

It was recently shown that treatment with UNC9994 decreased the viabilities of MMQ and GH3 cells [23] at very high doses, from 3.75 μM to 30 μM. Here, we used UNC9994 at the same dose as cabergoline (100 nM) and compared their effects in the same experiment.

Regarding hormone production, we found that the cabergoline and both biased agonists reduced PRL secretion with comparable efficacy. The MLS1547 effect on reducing PRL secretion was expected, since G-protein-mediated pathways regulate calcium and cAMP, the intracellular messengers that play major roles in controlling the fusion of secretory vesicles with the plasma membrane in order to release PRL [24], and since it is known that dopamine-induced inhibition of PRL release is affected by PTX treatment [25,26,27]. The effect of the UNC9994 on the PRL release can be explained by the observation that UNC9994 in Xenopus oocytes has activated G-protein-coupled inward rectifier potassium channels [28], which contributed to the control of PRL secretion [24]. The observation that both biased DRD2 ligands are effective in inhibiting PRL release is consistent with the redundancy of DRD2-mediated pathways that control PRL exocytosis, strengthening the blockade of basal PRL release [24].

To translate the results obtained in the rat cell line MMQ to a human model, we used primary cultures derived from surgically removed human PRL-PitNETs. We found that in cabergoline-responsive tumors, UNC9994 exerted similar effects on cell proliferation and PRL secretion, whereas in tumors resistant to cabergoline, it reached good inhibition of both cell growth and PRL release, suggesting that selective β-arrestin 2 pathway activation can overcome resistance to unbiased DRD2 agonists. Admittedly, the use of only two primary cultures of PRL-PitNET did not allow for broad generalizations. However, these data, combined with mechanistic data obtained for MMQ cells, strongly encourage further studies, in a large cohort of patients, to test the efficacy of UNC9994.

In addition, since UNC9994 has exerted antipsychotic-like activity in mice [17], it may represent an interesting, new and unique pharmacological therapy in patients, with PRL-PitNETs, as antipsychotic drugs for mental disorders. Indeed, in these patients, DAs are less effective and may, in some rare cases, give psychotic exacerbation due to the opposite effects of antipsychotics and DAs on DRD2 [29,30,31]. Moreover, the use of UNC9994 as an antipsychotic would avoid the risk of hyperprolactinemia, which can represent an adverse effect of conventional antipsychotics and some atypical antipsychotics.

We then compared the effects of DRD2 agonists in surgically removed human NF-PitNETs, since no cell line model of NF-PitNETs is available. We previously showed that DRD2 agonist BIM53097 induced a reduction in AKT phosphorylation in a subset of NF-PitNETs (about 37%), and that this ability was mediated by β-arrestin 2 and correlated to its antimitotic effects (observed in about 30% of tumors) [12]. Since in NF-PitNETs, the cAMP pathway exerts an antiproliferative effect, as in PRL-PitNET cells [14], and β-arrestin 2 is required for DRD2′s inhibitory effects on cell proliferation [12], we could expect to observe an increased effect of the UNC9994 vs. the cabergoline, as in the MMQ cells, and no effectiveness of the MLS1547. In contrast, we found that both unbiased and biased DRD2 ligands reduced cell proliferation to similar extents in 4/23 NF-PitNETs, whereas 13 of the 23 primary cultures were resistant to all DRD2 ligands. In the remaining six tumors, we observed great heterogeneity of responsiveness, including tumor responsiveness to only cabergoline and UNC9994, to only cabergoline and MLS1547 and to only cabergoline. Overall, the best result was obtained with cabergoline, which induced inhibitory effects in about 43% of tumors, as expected, in contrast with the 26% of tumors responsive to UNC9994. No correlations were found between ligand efficacy and lineage derivation, nor DRD2 and β-arrestin 2 transcript expression. It should be considered that the degree of UNC9994 agonist/antagonist activity in the β-arrestin 2 pathway depends on the expression levels of G-protein-coupled receptor kinase-2 (GRK2), which is directly recruited by DRD2 and mediates DRD2 phosphorylation, thus enhancing the affinity of the receptor to β-arrestins [32,33]. It has been shown that when GRK2 expression is low, UNC9994 behaves more as a β-arrestin-biased antagonist, and when GRK2 expression is high, UNC9994 gains agonist activity in the β-arrestin pathway [34]. We can hypothesize that low expression of GRK2 might be one possible cause of NF-PitNET unresponsiveness to UNC9994. Indeed, it has previously been shown that GRK2 was expressed in PRL-PitNETs at the highest levels compared with in GH- and NF-PitNETs [34].

The antiproliferative effects of MLS1547 in a subset of NF-PitNETs might be due to crosstalk between the cAMP and MAPK pathways, which, in the pituitary, contribute to regulation of cell proliferation and hormone secretion [15].

Overall, these data highlight the great heterogeneity of NF-PitNETs regarding their responses to DAs, as has been previously demonstrated [3,4,5,6,12]. Further studies are required to identify the alterations, probably common to the two pathways, responsible for DA resistance. Our results suggest that in this type of tumor, the selective activation of a single pathway cannot improve cabergoline efficacy or overcome resistance. However, since UNC9994 is a relatively low-potency ligand [17], we cannot exclude that the development of new, more potent biased agonists could improve the antitumoral effects elicited by DRD2 activation.

## 5. Conclusions

In conclusion, the preferential activation of the β-arrestin 2 signaling pathway by the biased DRD2 ligand UNC9994 enhances DRD2-mediated antiproliferative effects in PRL-PitNETs compared to cabergoline while maintaining a similar antisecretory activity, suggesting a new pharmacological approach for resistant or poorly responsive tumors. In contrast, this strategy is not beneficial in NF-PitNETs.

## Data Availability

Data available in a publicly accessible repository. The data presented in this study are openly available at https://dataverse.unimi.it/privateurl.xhtml?token=7dd1cd51-55bf-4a6f-a5de-8bf8fcb060e1 (accessed on 16 April 2023).

## Supplementary Materials

The following supporting information can be downloaded at: https://www.mdpi.com/article/10.3390/cancers15123218/s1. Table S1: Primers sequences are detailed. Table S2: Thermal cycling conditions are provided. Figure S1: DRD2 isoforms (D2L, D2S) and β-arrestin 2 expression in NF-PitNETs (*n* = 22). Figure S2: SF1 (a) and PIT1 (b) expression in NF-PitNET. File S1: Original blot.
