# Supplementary material for: A β-Arrestin 2-Biased Dopamine Receptor Type 2 (DRD2) Agonist Is More Efficacious Than Cabergoline in Reducing Cell Proliferation in PRL-Secreting but Not in Non-Functioning Pituitary Tumor Cells
Source: Cancers (Basel). 2023 Jun 16;15(12):3218. doi: 10.3390/cancers15123218 (PMC10296728; doi:10.3390/cancers15123218)
Supplement: Supplementary file 1 [file cancers-15-03218-s001.zip › cancers-2378280-supplementary.pdf]

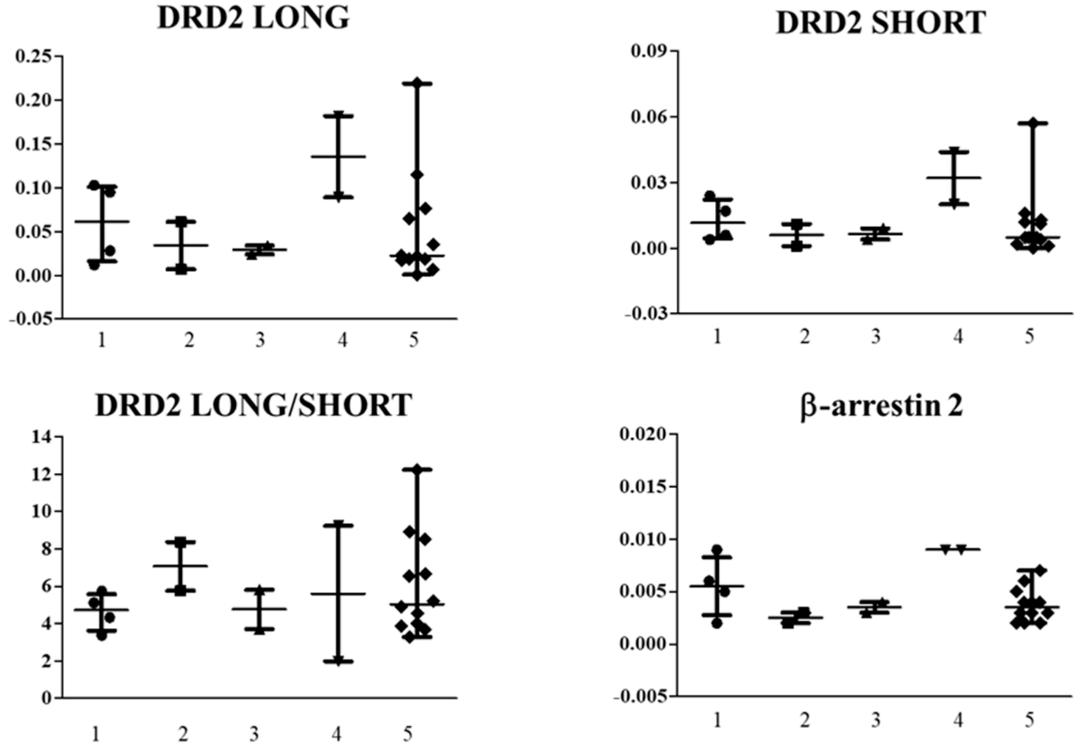

Supplementary figure S1. DRD2 isoforms (D2L, D2S) and β-arrestin 2 expression in NF-PitNETs (n=22). Receptors mRNA expression was evaluated in NF-PitNETs responsive to: cabergoline, UNC9994 and MLS1547 (1); cabergoline and UNC9994 (2); cabergoline and MLS1547 (3); cabergoline (4) or not responsive (5). The vertical scatter plots with median and interquartile range (IQR) show the different (D2L, D2S, and β-arrestin 2) mRNA relative expression in NF-PitNETs.

(a)

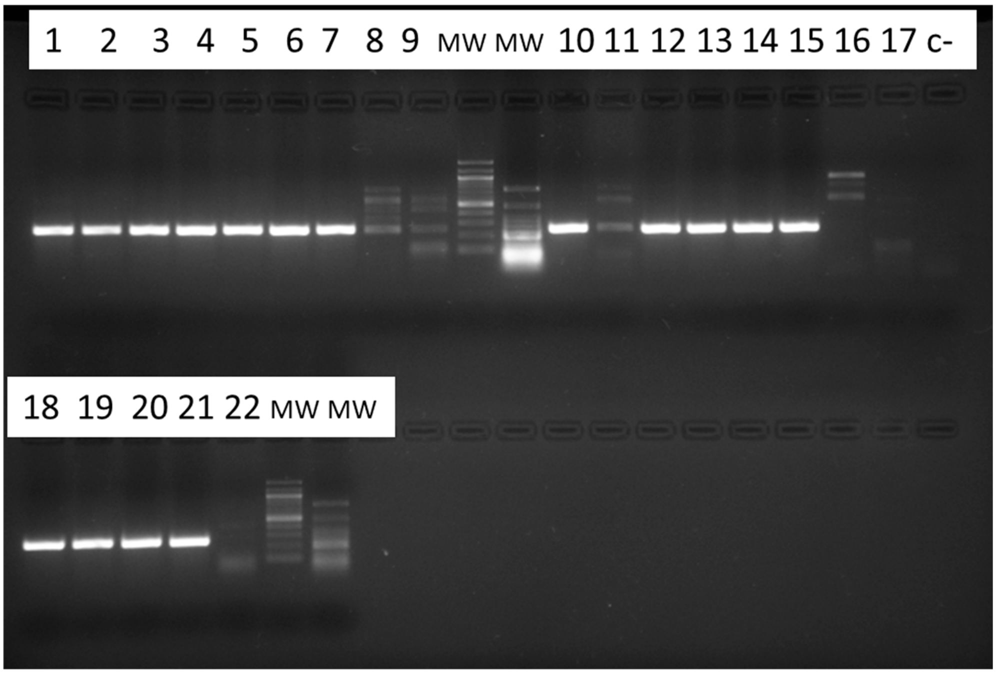

(b)

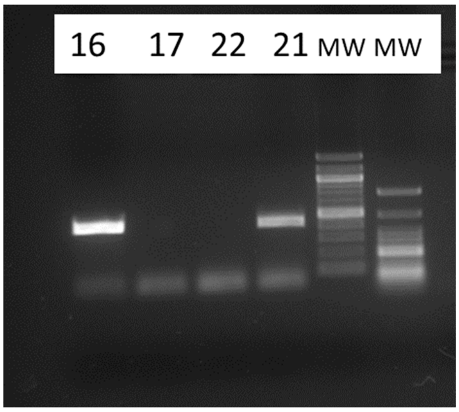

Supplementary figure S2. SF1 (a) and PIT1 (b) expression in NF-PitNET. RT-PCR analysis on NF-PitNET samples (n=22) to detect SF1 (a) and PIT1 (b) expression. MW= molecular weight.

Supplementary Table S1: primers sequences are detailed

Gene	Primer sequence	
SF1	forward:	5′-AGCTGCAAGGGCTTCTTCAA- 3′
	reverse:	5′-GAATCTGTGCCTTCTTCTGC- 3′
PIT1	forward:	5′-CCCAGAGCTGCTGATTTCAA- 3′
	reverse:	5′- GCCATCCTCATGATCTCTTG- 3′
DRD2 long	forward:	5′-CTCCACTAAAGGGCAACTGTACT- 3′
	reverse:	5′-TCCACTCTCCGCCTGTTCAC- 3′
DRD2 short	forward:	5′-TCATTGTCACCCTGCTGGTC- 3′
	reverse:	5′-GGGCAGCCTCCTTTAGTGG- 3′
β-arrestin 2	forward:	5′- GCTTCCAGCACCATCGTGAA- 3′
	reverse:	5′- CAGCTCCACAGAGA-CATCCC- 3′

Supplementary Table S2 : thermal cycling conditions are provided

Thermal cycling conditions for RT-PCR			
Stage 1	95 °C	2'	1x
Stage 2	95 °C	30"	40x
	57 °C	30"	
	72 °C	30"	
Stage 3	72 °C	5'	1x
Stage 4	12 °C	∞	1x
