# Supplementary material for: A β-Arrestin 2-Biased Dopamine Receptor Type 2 (DRD2) Agonist Is More Efficacious Than Cabergoline in Reducing Cell Proliferation in PRL-Secreting but Not in Non-Functioning Pituitary Tumor Cells
Source: Cancers (Basel). 2023 Jun 16;15(12):3218. doi: 10.3390/cancers15123218 (PMC10296728; doi:10.3390/cancers15123218)
Supplement: Supplementary file 1 [file cancers-15-03218-s001.zip › File S1.pdf]

Western blots of CD3, GAPDH - Fig. 1b

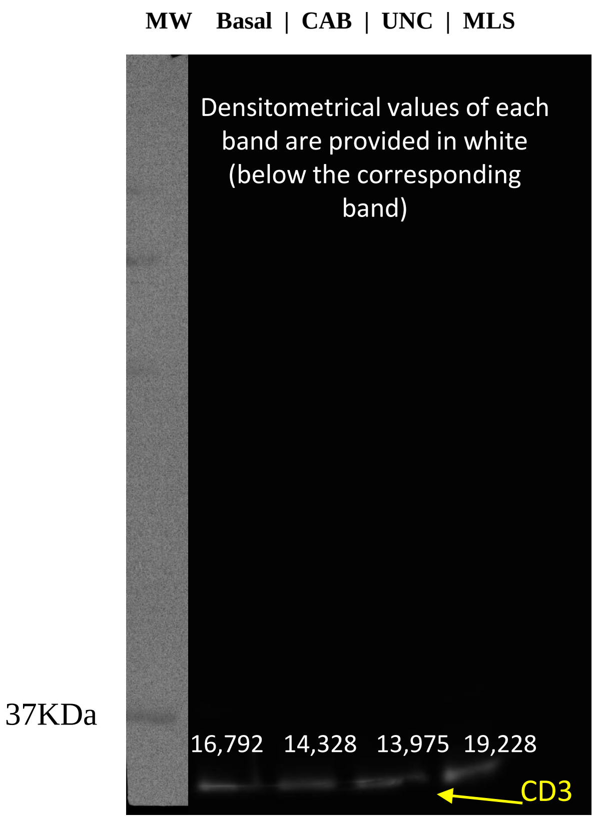

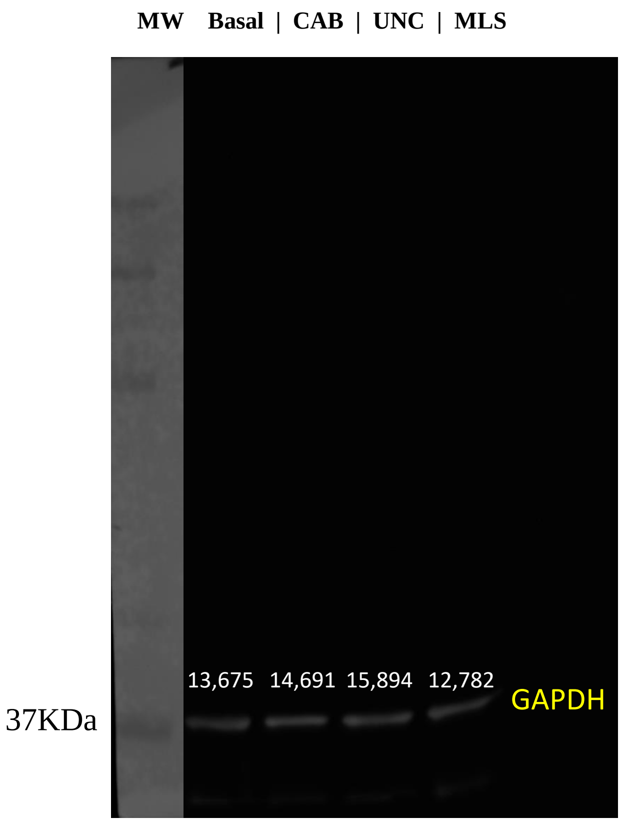

The molecular weight have been acquired in white light and juxtaposed at the original position of western blot bands

Western blots of pAKT, totAKT - Fig. 1e

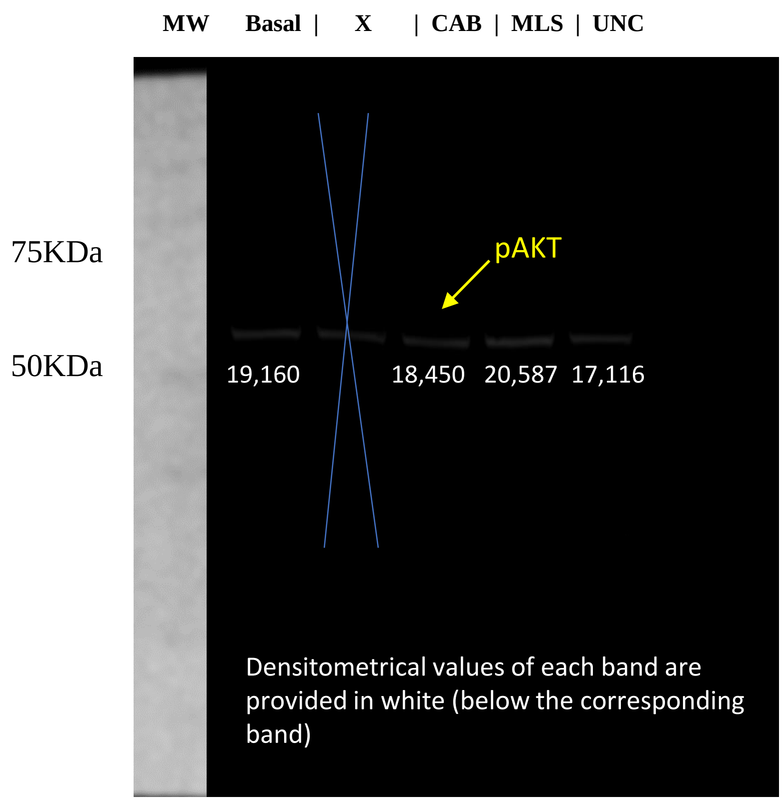

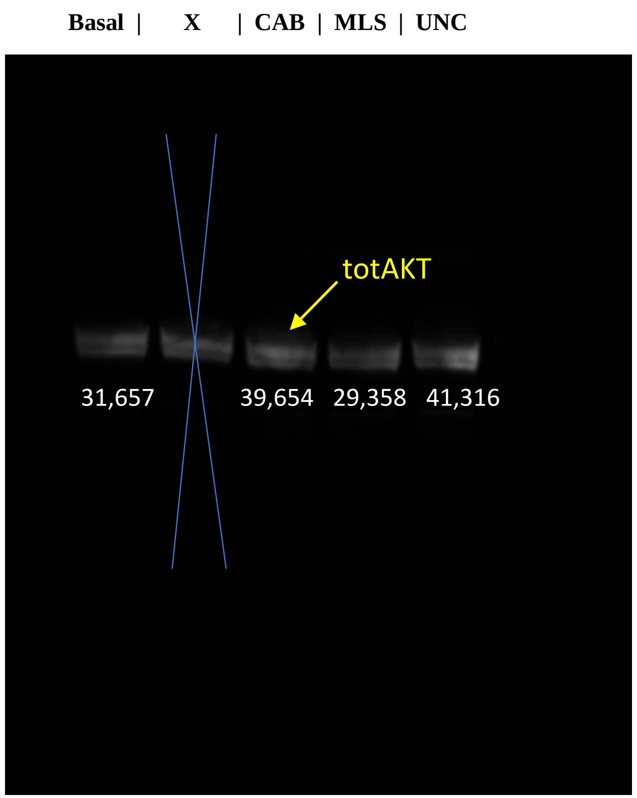

Densitometrical values of each band

The molecular weight have been acquired in white light and juxtaposed at the original position of western blot bands

The molecular weight have not been acquired for this blot (no aspecific bands are usually detected with the above mentioned antibodies)

Western blots of pAKT, totAKT - Fig. 1g

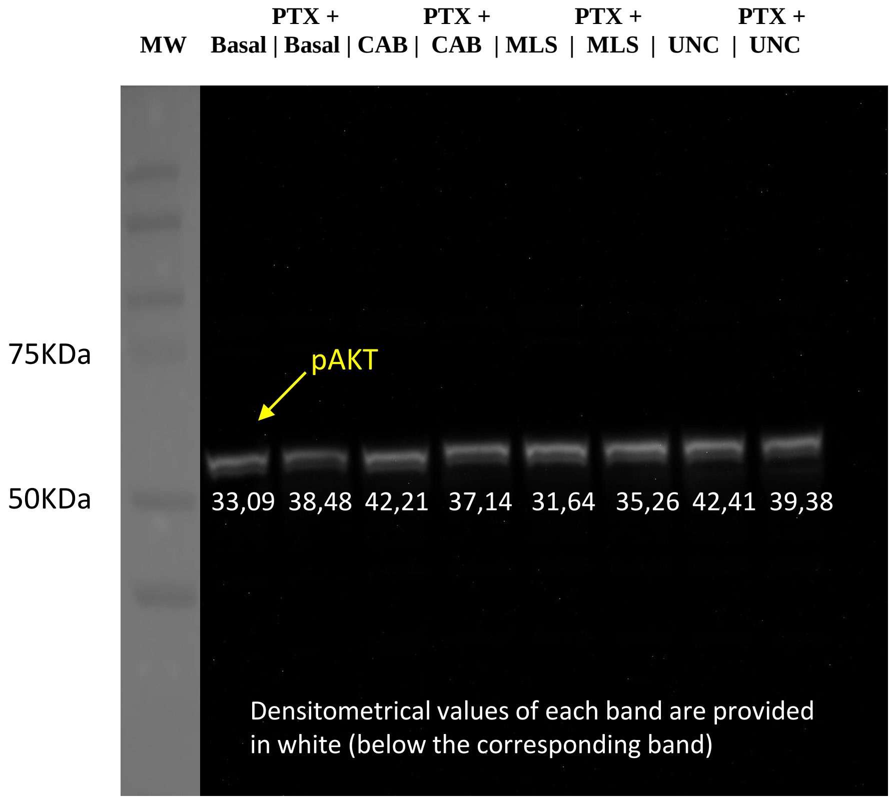

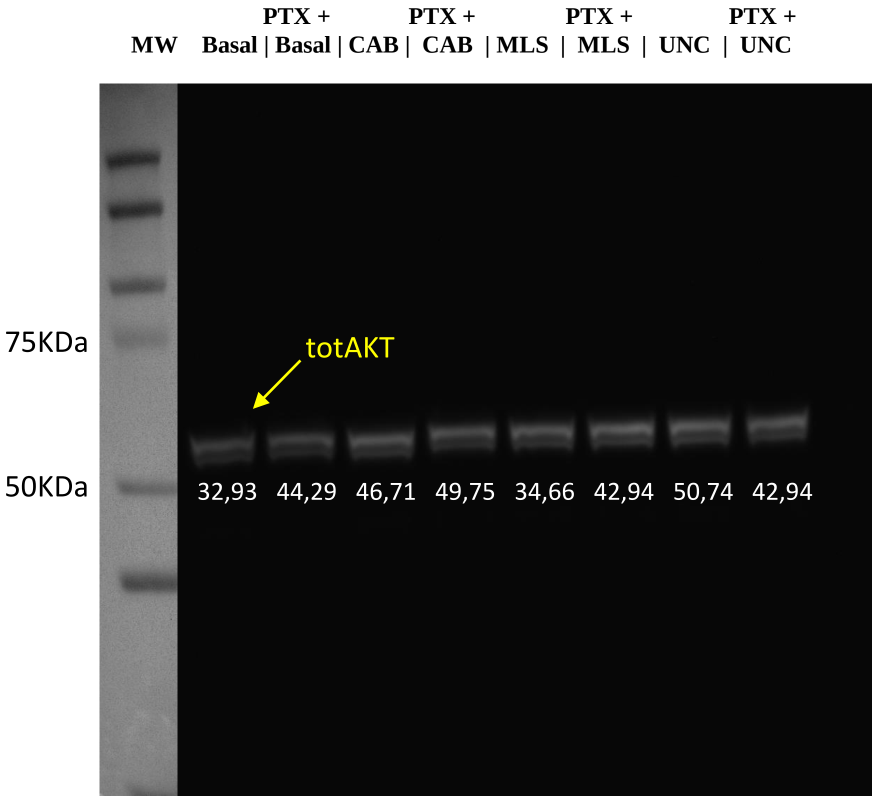

The molecular weight have been acquired in white light and juxtaposed at the original position of western blot bands
